# Anesthetic Challenges of a Patient With Limb-Girdle Muscular Dystrophy in a Patient With Colon Cancer

**DOI:** 10.7759/cureus.69009

**Published:** 2024-09-09

**Authors:** Austin Smith, Cindy Yeoh, Christopher Massengill, Wendy Yang, Amreesh Mahil

**Affiliations:** 1 Department of Anesthesiology, Moffitt Cancer Center, Tampa, USA; 2 Morsani College of Medicine, University of South Florida Morsani College of Medicine, Tampa, USA

**Keywords:** difficult airway management, limb-girdle muscular dystrophy, malignant hyperthermia, musculoskeletal, ryr1 gene

## Abstract

Limb-girdle muscular dystrophy (LGMD) presents a unique challenge for anesthesiologists because of the potential complications related to surgery and anesthesia. This is a case of a 55-year-old male with colon cancer and a history of LGMD, who underwent a low anterior resection colectomy under general anesthesia. Because of the pathogenic variants in the RYR1 gene implicated in various congenital myopathies, we review clinical concerns associated with LGMD and describe the anesthetic management of our patient with LGMD and a potentially difficult airway.

## Introduction

Limb-girdle muscular dystrophy (LGMD) encompasses a heterogeneous group of genetic disorders characterized by progressive weakness and wasting muscles, particularly affecting the pelvic and shoulder girdles [1]. It is among the most common forms of muscular dystrophy, with both autosomal dominant and autosomal recessive inheritance patterns identified [2]. Over 30 LGMD types have been identified, being broadly classified as LGMD type I (autosomal dominant) and type II (autosomal recessive). LGMDs are heterogenous in their symptomatology, varying widely in age of presentation, muscle involvement, disease course, as well as in genetic penetrance and expressivity [2]. The clinical presentation of LGMD varies widely, ranging from childhood-onset with severe muscle weakness, to late-onset with preserved ambulation into adulthood. Clinical manifestations commonly involve the musculoskeletal, respiratory, and cardiovascular systems and can also present as a systemic disorder involving the central nervous system [3,4]. These patients present the anesthesiologist with challenges that include respiratory insufficiency due to muscle weakness, intubation difficulties from distorted anatomy, increased risk for pulmonary aspiration, and arrythmias related to cardiomyopathy. Patients with LGMD who have general anesthesia with volatile inhalational anesthetics and succinylcholine are also at increased risk of malignant hyperthermia (MH) and rhabdomyolysis, prompting consideration of total intravenous anesthesia and avoidance of MH triggers.

We present the anesthetic management of a patient with LGMD and a difficult airway, a known challenge associated with LGMD, yet rarely described in the literature.

## Case presentation

A 55-year-old male with colon cancer presented for a low anterior resection. The patient had a medical history significant for LGMD with no prior exposure to anesthesia. The patient’s symptoms predominantly affected his upper extremities in the shoulders. A detailed pre-anesthesia examination was done in the Pre-Anesthesia Testing Clinic a few weeks before the surgery. All the routine blood tests were done and within normal limits.

On examination, there was concern for a difficult airway caused by the patient’s mouth opening of <2 cm and a beak-shaped mouth and jaw. While difficult airways are not typically associated with LGMD, the plan was to proceed with caution and perform an awake fiberoptic intubation to secure the airway.

The anesthesia machine and equipment were prepared for an MH-susceptible patient. A new anesthesia machine with fresh circuits and CO2 absorbers was used and all vaporizers were removed. While the operating room was being prepared, the patient’s airway was topicalized with nebulized 4% lidocaine. Before proceeding to the operating room, dexmedetomidine was started at an infusion rate of 0.7 mcg/kg/min, and 0.2 mg of glycopyrrolate was also administered to reduce secretions. In the operating room, the patient was positioned at 45 degrees and semireclined. The airway was secured under direct fiberoptic visualization with a 7.0 mm endotracheal tube. Maintenance of anesthesia was achieved with total intravenous anesthesia (TIVA) with propofol (100 mcg/kg/min), dexmedetomidine (0.7 mcg/kg/min), fentanyl (1-2 mcg/kg), and paralysis was achieved with rocuronium (0.6 mg/kg). The patient was managed conservatively in the postoperative period and extubated without incident the following day in the intensive care unit. No new neurological deficits were noticed at the time of his discharge.

## Discussion

LGMD presents a unique challenge in the context of anesthesia and airway management because of its potential complications, including respiratory muscle weakness and compromised airway patency. In this case report, we highlight the successful use of TIVA for avoiding potential MH triggers and awake-fiberoptic intubation as a safe and effective technique for securing the airway in a patient with LGMD.

One of the key considerations for managing patients with LGMD is the risk of respiratory compromise, particularly during introduction of anesthesia when muscle relaxation can further exacerbate existing respiratory weakness. Awake-fiberoptic intubation offers several advantages in this setting. By preserving spontaneous ventilation, it minimizes the risk of respiratory decompensation associated with muscle relaxation, allowing for a controlled and gradual approach to securing the airway. This is particularly important in patients with LGMD, in which rapid deterioration in respiratory function can occur. Furthermore, awake-fiberoptic intubation provides the opportunity for thorough airway assessment and planning before induction of anesthesia. In our case, careful evaluation of the patient’s airway anatomy revealed potential challenges, including limited mouth opening, which could have complicated conventional intubation techniques. By performing the procedure while the patient was awake and cooperative, we were able to anticipate these difficulties and tailor our approach accordingly, ensuring a smooth and successful intubation without needing rescue maneuvers or alternative airway devices.

If appropriate for the type of surgery, laryngeal mask airway with or without muscle relaxants can be used as an alternative to endotracheal intubation in patients with airway involvement of LGMD. Additional considerations include temperature and invasive blood pressure monitoring, with interval lab analysis of lactate, blood ion, and creatine kinase levels to detect muscle damage [2].

**Figure 1:**
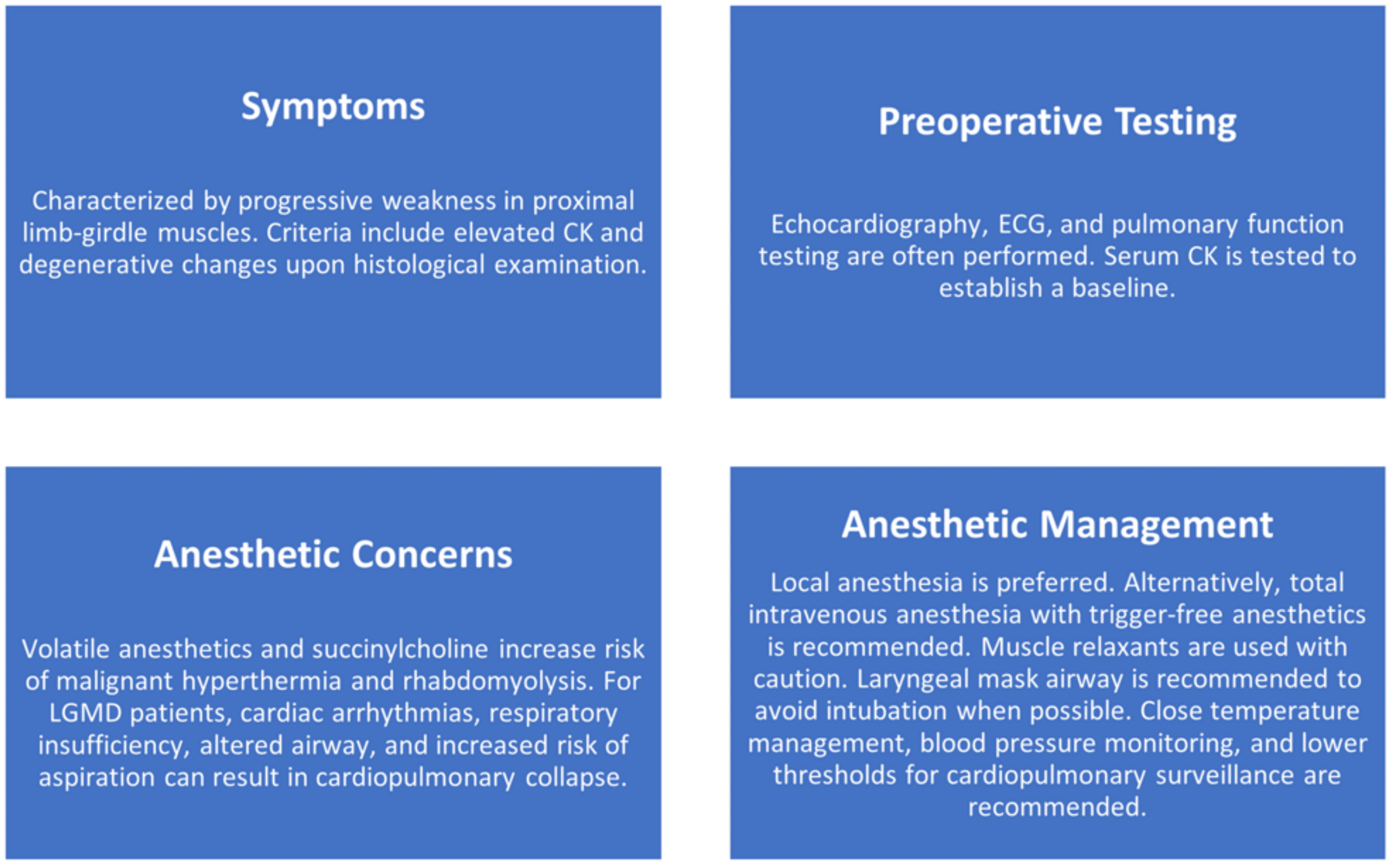
Characterization and anesthetic management of LGMD. Abbreviations: CK, creatine kinase; ECG, electrocardiogram; LGMD, limb-girdle muscular dystrophy. Image Credit: Dr. Mahil [2,5,6]

Inhaled anesthetics and succinylcholine are contraindicated for patients with LGMD because of an increased likelihood of developing rhabdomyolysis and hyperkalemia [2]. In almost all patients, creatinine kinase levels are elevated because of breakdown of muscle fibers [4]. Thus, neuraxial or local anesthesia is preferred to general anesthesia when possible [5]. Using TIVA avoids the potential triggers of MH. In our case, we maintained appropriate anesthetic depth with infusions of propofol and dexmedetomidine, in addition to intermittent boluses of fentanyl. Rocuronium, a nondepolarizing neuromuscular blocker was adequately titrated to keep the patient at 0/4 twitches during the procedure. Before emergence, the patient was reversed with sugamaddex at 4 mg/kg. Dantrolene was available in case of suspected MH but was kept in the pharmacy on the same floor as the operating room. Bispectral index brain monitoring was used in the setting of TIVA to ensure an adequate depth of anesthesia. The susceptibility of patients with muscular dystrophy to sedatives, anesthetics, and neuromuscular blocking agents may result in perioperative cardiopulmonary complications and prolonged recovery (Figure 1) [2,5,6], and should therefore be considered when devising an anesthetic plan [7].

## Conclusions

LGMD presents unique challenges to the anesthesiologist because of the severity of muscle weakness and potential for respiratory compromise. Volatile anesthetics and succinylcholine should be avoided because of the risk of malignant hyperthermia and rhabdomyolysis. If LGMD is associated with distorted anatomy and difficult airway concerns, the anesthesiologist should consider an awake fiberoptic intubation. Intraoperative monitoring of hemodynamics and temperature should be maintained for LGMD patients under general anesthesia because of cardiopulmonary complications and malignant hyperthermia risks. With careful preparation and planning, general anesthetics can be performed safely in this patient population.

## References

[1] Iyadurai SJ, Kissel JT (2016). The limb-girdle muscular dystrophies and the dystrophinopathies. Continuum (Minneap Minn).

[2] Angelini C (2020). LGMD. Identification, description and classification. Acta Myol.

[3] Liang WC, Jong YJ, Wang CH (2020). Clinical, pathological, imaging, and genetic characterization in a Taiwanese cohort with limb-girdle muscular dystrophy. Orphanet J Rare Dis.

[4] Murphy AP, Straub V (2015). The classification, natural history and treatment of the limb girdle muscular dystrophies. J Neuromuscul Dis.

[5] Schieren M, Defosse J, Böhmer A, Wappler F, Gerbershagen MU (2017). Anaesthetic management of patients with myopathies. Eur J Anaesthesiol.

[6] Cao XQ, Joypaul K, Cao F, Gui LL, Hu JT, Mei W (2019). Anesthetic management of a patient with limb-girdle muscular dystrophy 2B:CARE-compliant case report and literature review. BMC Anesthesiol.

[7] Sarkılar G, Mermer A, Yücekul M, Çeken BM, Altun C, Otelcioğlu Ş (2014). Anaesthetic management of a child with limb-girdle muscular dystrophy. Turk J Anaesthesiol Reanim.

